# Detection of two zoonotic pathogens, *Seoul orthohantavirus* and pathogenic *Leptospira*, in rats of Bamako, Mali (2021−2023)

**DOI:** 10.1016/j.onehlt.2025.101085

**Published:** 2025-05-23

**Authors:** J. Garona, A. Berard, C. Tatard, A. Kwasiborski, P. Gauthier, S. Ag Atteynine, V. Hourdel, A. Eusebe, C. Diagne, V. Caro, C. Brouat, N. Charbonnel, V. Sauvage, L. Granjon, G. Castel

**Affiliations:** a CBGP, IRD, INRAE, CIRAD, Institut Agro, Univ Montpellier, Montpellier, France; b Institut Pasteur, Université Paris Cité, Environment and Infectious Risks Unit, Laboratory for Urgent Response to Biological Threats (CIBU), Paris, France; c Institut d'Economie Rurale, IER, Bamako, Mali; d Institut Pasteur, Université Paris Cité, Environment and Infectious Risks Unit, Centre National de Référence des Hantavirus, Paris, France

**Keywords:** *Seoul orthohantavirus*, *Leptospira*, Rat-borne pathogens, Urban ecosystem

## Abstract

*Seoul orthohantavirus* (SEOV) and pathogenic leptospires, two zoonotic agents causing similar symptoms in humans, were investigated in rat populations across several neighborhoods in Bamako, Mali. SEOV seroprevalence in brown rats (*Rattus norvegicus*) reached 14.8 %, while no infection was detected in black rats (*Rattus rattus)*. Pathogenic leptospires were found in 8.7 % of brown rats, with significant inter-neighborhood variations, while only one black rat tested positive. Viral genetic analyses suggested that SEOV strains circulating in Bamako may result from a reassortment between two SEOV lineages. These findings highlight the widespread distribution of SEOV and the localized presence of pathogenic leptospires in Bamako, emphasizing the role of brown rats as reservoirs. These results can guide municipal authorities in implementing rodent control and prevention strategies to mitigate associated public health risks in Bamako.

## Highlights

• Seoul virus and pathogenic leptospires were detected in brown rat populations from Bamako city.
• Seoul virus is prevalent in most neighborhoods of Bamako.
• The distribution of leptospires varies significantly across the city.
• Seoul virus strains of Bamako could result from a reassortment between two lineages.

## 1 Introduction

Urban areas are widely acknowledged to serve as major entry points for non-native species due to their connection to the global transportation network. Moreover, urban conditions and the intensity of human activities may facilitate survival, reproduction, and spread of non-native species that thrive in close association with humans [1,2] some of them having become major invasive species in many regions worldwide [3]. Among these, rodents are particularly noteworthy [4]. Beyond their massive ecological and economic impacts, rodents are also key reservoirs of zoonotic pathogens responsible for over 400 million human infections annually [5]. Many of these diseases have non-specific symptoms and can be confounded with diseases like malaria in Africa, especially when healthcare workers are unaware of the local circulation of pathogens [6]. This is particularly true for *Seoul orthohantavirus* (SEOV) induced diseases and for leptospirosis, which are still often misdiagnosed due to frequently mild and atypical clinical manifestations [7,8]. Moreover, these two agents can both cause acute renal failure [9], which can complicate differential diagnosis between them and lead to mistreatment [[10], [11], [12]].

SEOV (order *Elliovirales*: family *Hantaviridae*) is an enveloped virus with a tri-segmented single-stranded negative-sense RNA genome, primarily hosted by *Rattus norvegicus* but also by *Rattus rattus*. SEOV infection is chronic and nearly asymptomatic in rats [13]. SEOV is transmitted to humans via aerosols contaminated with rat excretions and can cause hemorrhagic fever renal syndrome (HFRS), with ∼1 % lethality [14]. Due to the global distribution of its rodent hosts, SEOV is present worldwide [7]. However, it was only recently unambiguously identified in Africa through sequencing, specifically in Senegal and Benin [15,16]. While serological evidence already suggests orthohantavirus-associated human disease in Africa [17,18], notably in Mali [19], increased continental and intercontinental trade may accelerate SEOV spread and number of human cases on this continent.

Leptospirosis, caused by spirochetes of the genus *Leptospira,* affects at least one million people annually with 60,000 deaths [20]. Leptospires circulate in various mammals, particularly rodent species, which are chronic asymptomatic carriers, shedding bacteria through urine [21]. Transmission occurs via direct contact with infected hosts or, more commonly, indirectly through contaminated environments, with rainfall events facilitating exposure [22]. Leptospires could survive in the environment for months [23]. Patients can develop severe illness, including acute renal failure [23]. In Sub-Saharan Africa, leptospirosis is recognized as an important zoonosis [24]. Recent data on pathogenic leptospires in rodents in Benin [25] and Niger [26], have been published. Data from Mali remain sparse, but a retrospective analysis of serum samples from acutely ill patients [19] suggests underestimated pathogenic leptospire circulation.

Since leptospires and orthohantaviruses share the same rodent host species, co-infections can occur, as reported in several countries [27,28]. The impact of such co-infections on host health and on pathogenicity of either pathogen remains unclear. However, they may increase the likelihood of human infection with both pathogens [19] and potentially lead to more severe disease outcomes [29].

Bamako, Mali's capital, had an estimated population of 4.2 million in 2022 and continues to grow. The introduction of *Rattus* species can be traced back to the early 20th century, coinciding with the city's development and the establishment of rail transportation between Senegal and Mali that is considered to have represented the main channel of invasion of Bamako by *Rattus* species. Bamako presents risk factors for the introduction and circulation of SEOV and pathogenic leptospires among *Rattus* species. Its socio-economic situation and trade connections with countries where SEOV is already present, heighten its vulnerability to SEOV introduction. The Niger river that flows through the city, the market gardening areas scattered across it, and the complex open sewage systems running through it provide a priori favorable habitats for the circulation of pathogenic leptospires within *R. norvegicus* and *R. rattus* populations, particularly during the rainy season, which lasts from May to October.

In this context, our study aims to investigate the presence and distribution of SEOV and pathogenic leptospires in *Rattus* species, capitalizing on extensive small mammal surveys conducted in Bamako between 2021 and 2023. These investigations are crucial for providing valuable data to inform public health strategies and improve the management of potential zoonotic threats in an urban area, where previous studies have shown that up to 20.4 % of febrile disease cases in hospitals remain unexplained [[30], [31], [32]].

## 2 Materials and methods

### 2.1 Sampling

Rodents were collected from homes across 16 neighborhoods throughout Bamako (Fig. S1 in supplementary material 1), between 2021 and 2023 during the “Rainy” (May–October), “Cool dry” (November–February), or “Hot dry” (March–April) seasons (Table S1 in supplementary material 5). Average climatic values of each season between 1991 and 2024 are provided (Table S2 in supplementary material 6).

Trapping sessions followed standardized procedures [33], pre-approved by the Malian National Directorate of Water and Forests and the Ethics Committee of the Malian National Institute of Public Health (Decision n°18/2021/CE-INSP), and meeting Nagoya protocol requirements. Live trapping lasted 4–5 nights, totaling 450–600 trap-nights per neighborhood. Two types of traps were used: locally made wire-mesh live traps and Sherman folding box traps, set (in pairs) inside housing or working units including inner yards, between one and three consecutive nights. They were checked and (re)baited once a day with peanut butter spread on a slice of fresh onion. Captured individuals were euthanized by cervical dislocation, as recommended by [34], then measured, weighed, sexed, and classified by sexual maturity according to classical criteria (position / size of testicles and development of seminal vesicle in males, vaginal opening and presence of embryos or placental scars in females). Organ samples (spleen, kidney and digestive tracts) were stored in ethanol and blood was collected on Whatman paper. For some individuals trapped in Missabougou and Fadjiguila neighborhoods, lungs were stored in DNA/RNAShield. Details of trapping methods and data on small mammals caught during this study are openly available in the DataSuds repository [35]. The associated biological samples are now part of the CBGP reference collection of small mammals [36].

### 2.2 Molecular analyses for the detection of pathogenic leptospires

DNA was extracted from ethanol-preserved kidney tissue of each rodent using the 96-Well Plate Animal Genomic DNA Mini-Preps Kit (Bio Basic Inc.) and eluted with 200 μL of elution buffer. Detection of pathogenic leptospires was performed using a real-time PCR approach targeting a fragment of the *lipL32* gene as described in [26]. All experiments were conducted in duplicate using a LightCycler® 480 instrument (Roche Diagnostics). qPCR positive controls (DNA extracted from *Leptospira interrogans* culture) as well as extraction and qPCR negative controls were added to each reaction plate.

### 2.3 Serological analysis for the detection of orthohantaviruses

The presence of anti-*Orthohantavirus* IgG in rats was detected by ELISA tests using dried blood punches (6 mm) of Whatman paper, eluted in PBS 1× tween 0.1 % (overnight at room temperature). 25 ng of recombinant Murinae-associated *Orthohantavirus* nucleoprotein antigen (HTV-001, Prospec) or 25 ng of a negative antigen (LSV-002, Prospec) was adsorbed to POLYSORP plate (overnight at 4 °C). 5 μL of each eluate sample was incubated with these 2 antigens. One positive and one negative eluate were used as controls for each plate. A peroxidase-coupled rabbit anti-rat IgG (20 μg.mL-1) was used as secondary antibody. Absorbance was then measured at 450 and 620 nm. Samples with a corrected optical density (OD) value (∆OD antigen HTV - ∆OD negative antigen) greater than 0.1 were considered seropositive.

### 2.4 Viral RNA extraction, real-time RT-PCR and sequencing of SEOV

RNA extraction was performed on lung tissues of seropositive rats using the NucleoSpin Dx Virus kit (Macherey-Nagel, Germany). Samples were tested using a SEOV-specific real-time RT-PCR according to [37], with minor modifications. Primer sequences were forward: 5’-CAT GGC WTC HAA GAC WGT GGG-3′ and reverse: 5’-TTK CCC CAG GCA ACC AT-3′. Probe sequence was (VIC/MGB): 5’-TCA ATG GGR ATA CAA CT-3′. Whole-genome sequences of SEOV were then generated by an in-house amplicon-based nanopore sequencing method. The sequencing library was prepared using Native Barcoding Kit 24 V14 (SQK-NBD114.24) according to the manufacturer's recommendations and loaded onto FLO-MIN114 (R10.4.1) flow-cell for 12 h on a Mk1C MinION (O) device. The bioinformatics protocol proposed by the ARTIC Network (available at [38]) was adapted for data analysis and consensus sequence generation. For basecalling, de-multiplexing, mapping and consensus generation, the pipeline used the following tools: Guppy (v6.0), Minimap2 (v2.17-r941) and Medaka (v1.0.3), respectively. Sequences were deposited in Genbank under the accession numbers (PQ932549-PQ932563).

### 2.5 Phylogenetic analyses of orthohantaviruses sequences

Phylogenetic analyses were performed as described in [39] on three datasets composed of complete (or nearly complete) coding regions of S, M and L segment sequences of SEOV recovered in this study and of sequences representative of known SEOV lineages, available in GenBank. Thailand (THAIV), Hantaan (HTNV) and Dobrava (DOBV) orthohantaviruses were used as outgroups. A Maximum Likelihood method with a GTR + R model was applied for all datasets. Estimates of base and amino acid differences per site were calculated using a function implemented in MEGA (v11.0) with default parameter settings and ambiguous positions removed for each sequence pair.

### 2.6 Statistical analyses

We performed a model selection procedure following the approach described in [40] using the MuMIn and DHARMa packages in R software (v4.4.0). Analyses were restricted to the *R. norvegicus* dataset due to the limited sample size and low prevalence rates observed for *R. rattus* (see Results). Generalized Linear Models (GLMs) with a binomial distribution and logit link were used to model the likelihood of infection by SEOV (seropositive = 1; seronegative = 0) or *Leptospira* (positive = 1; negative = 0). Predictors included host traits (sex, weight), neighborhood variables (Table 1), and their two-way interactions. To assess potential non-random co-occurrence of both pathogens within individual hosts (i.e., co-infection), the presence/absence of each pathogen was included as an additional predictor (i.e., *Leptospira* status in the SEOV model and vice versa). Sexual maturity was added to *Leptospira* models to account for developmental stages, but omitted from SEOV models as immature rats (*N* = 182) were excluded to reduce maternal antibody bias.

**Table 1:** Percentage of (sero)postive *R. norvegicus* for *Orthohantavirus* and pathogenic *Leptospira* spp. for each neighborhood. The number of coinfected brown rats is provided. M/F: male/female; I/M: immature/mature; NT: not tested.

Neighborhood	*R. norvegicus* (number)	M/F ratio	I/M ratio	Orthohantavirus seroprevalence (%)	Leptospires prevalence (%)	Co-infections (number)
Badalabougou	25	1.3 (14/11)	0.3 (8/17)	8	20	1
Bamako Coura	27	0.69 (11/16)	1.25 (15/12)	22.22	3.7	0
Fadjiguila	44	1 (22/22)	0.47 (14/30)	20.45	0 (14 NT)	0
Hippodrome	23	0.21 (4/19)	0.92 (11/12)	8.69	0	0
Kalabanbougou	13	1.6 (8/5)	0.3 (3/10)	23.07	15.38	0
Lafiabougou	41	1.16 (22/19)	1.73 (26/15)	0	0	0
Missabougou	39	0.86 (18/21)	0.22 (7/32)	25.64	0 (17 NT)	0
Niamakoro	25	0.25 (5/20)	0.56 (9/16)	12	12	0
Niaréla	26	0.63 (10/16)	0.86 (12/14)	11.58	3.84	0
Ouolofobougou	19	0.73 (8/11)	0.58 (7/12)	21.01	0	0
Sabalibougou	27	0.69 (11/16)	0.27 (7/20)	29.62	3.7	0
Sotuba	17	0.89 (8/9)	1.13 (9/8)	0	0	0
Sokorodji	12	0.2 (2/10)	1 (6/6)	0	0	0
Bacojicoroni	20	0.67 (8/12)	0.67 (8/12)	20	45	3
Médina-Coura	18	0.38 (5/13)	0.29 (4/14)	22.22	27.77	2
Banconi	37	1.18 (20/17)	0.54 (13/24)	8.11	20.83 (13 NT)	1
Total	413	0.74 (176/237)	0.63 (159/254)	14.76	8.67	7

## 3 Results

### 3.1 Sampling

Brown rats trapped per neighborhood ranged from 12 individuals in Sokorodji to 44 in Fadjiguila (413 individuals in total; Table 1). Black rats were captured in only eight of the 16 sampled neighborhoods, with the number of individuals ranging from two in Missabougou, Bamako Coura and Médina-Coura to 11 in Kalabanbougou (42 individuals in total; Table 2). The overall sex-ratio was significantly unbalanced in brown rats (0.74; 176 males vs 237 females, Chi-2, *p* = 0.04), and at equilibrium in black rats (0.91; 20 males vs 22 females, respectively; Chi-2; *p* > 0.05). In both species, the proportion of immature individuals was significantly lower than that of mature individuals (159 vs 254 in brown rats, Chi-2, *p* = 8 × 10^−4^; 7 vs 35 in black rats, Chi-2, *p* = 4 × 10^−5^).

**Table 2:** Percentage of (sero)postive *R. rattus* for *Orthohantavirus* and pathogenic *Leptospira* spp. for each neighborhood. The number of coinfected brown rats is detailed per neighborhood. M/F: male/female; I/M: immature/mature; NT: non tested.

Neighborhood	*R. rattus* (number)	M/F ratio	I/M ratio	Orthohantavirus seroprevalence (%)	Leptospires prevalence (%)	Co-infections (number)
Bamako Coura	2	1 (1/1)	1 (1/1)	0	0	0
Kalabanbougou	11	1.2 (6/5)	0.1 (1/10)	0	0	0
Missabougou	2	1 (1/1)	0 (0/2)	0	0	0
Niaréla	4	1 (2/2)	0.33 (1/3)	0	0	0
Ouolofobougou	10	1 (5/5)	0.43 (3/7)	0	10	0
Sotuba	3	0 (0/3)	0 (0/3)	0	0	0
Bacojicoroni	8	0.6 (3/5)	0 (0/8)	0	0	0
Médina-Coura	2	- (2/0)	1 (1/1)	NT	NT	NT
Total	42	0.91 (20/22)	0.2 (7/35)	0	3	0

### 3.2 Prevalence of leptospires in *R. norvegicus* and *R. rattus*

**Fig. 1:**
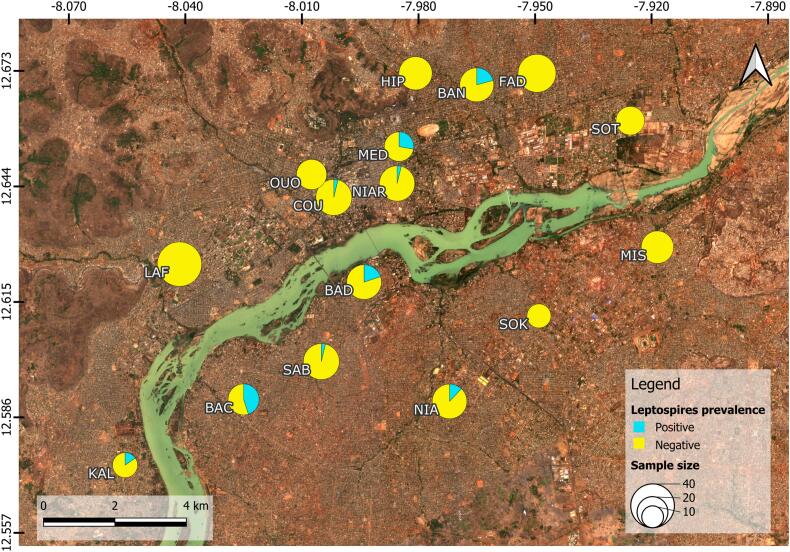
Prevalence of pathogenic leptospires by neighborhood for the brown rats. The size of the circles is proportional to the number of rats tested. Percentages of infected and non-infected rats are represented in blue and yellow respectively. GPS coordinate grid is indicated. The map was produced using QGis software (v3.28.1), and the base map was taken from 4 Sentinel-2 images (01–2024) downloaded from “theia_land.fr“. Badalabougou (BAD), Bamako Coura (COU), Fadjiguila (FAD), Hippodrome (HIP), Kalabanbougou (KAL), Lafiabougou (LAF), Missabougou (MIS), Niamakoro (NIA), Niaréla (NIAR), Ouolofobougou (OUO), Sabalibougou (SAB), Sotuba (SOT), Sokorodji (SOK), Bacojicoroni (BAC), Médina-Coura (MED), Banconi (BAN). (For interpretation of the references to colour in this figure legend, the reader is referred to the web version of this article.)

Out of the 369 brown rats tested (no kidney available for 44/413), 32 were found to be positive for pathogenic leptospires using the *lipL32* qPCR (CT values ranged from 20 to 40). The average citywide prevalence was 8.70 %, with neighborhood-level values ranging from 0 to 45 % (Table 1). The distribution of pathogenic leptospires was not homogeneous across Bamako, with half of the neighborhoods showing no presence (Fig. 1).

Out of the 40 black rats tested (no kidney available for 2/42), only one was found to be positive for pathogenic leptospires (prevalence of 2.5 %). It was sampled in the Ouolofobougou neighborhood (Table 2).

### 3.3 Seroprevalence of orthohantaviruses in *R. norvegicus* and *R. rattus*

**Fig. 2:**
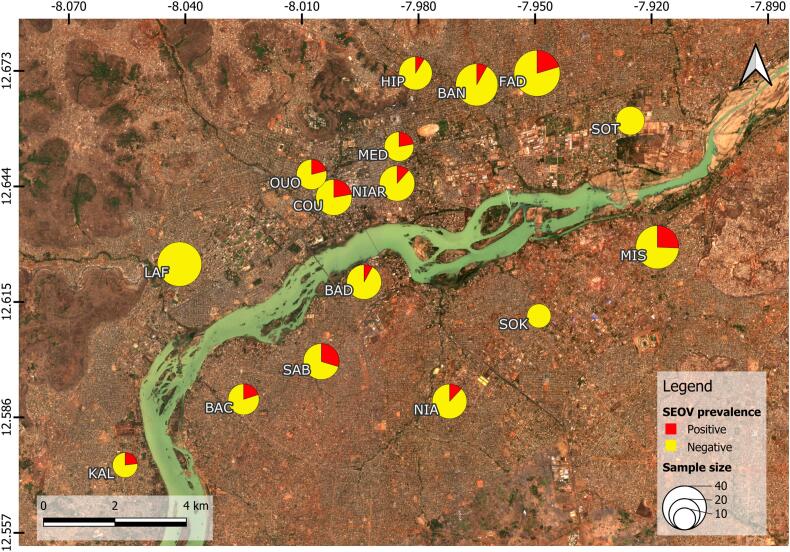
Seroprevalence of Murinae-associated orthohantavirus (anti-orthohantavirus IgG) in *R. norvegicus* from different neighborhoods of Bamako. Serological status was assessed using ELISA test performed on 413 brown rats trapped in Bamako between 2021 and 2023. The size of the circles is proportional to the number of rats tested. The colors red and yellow respectively represent the percentages of seropositive and seronegative brown rats. GPS coordinate grid is indicated. The map was produced using QGis software (v3.28.1), and the base map was taken from 4 Sentinel-2 images (01–2024) downloaded from “theia_land.fr“. Badalabougou (BAD), Bamako Coura (COU), Fadjiguila (FAD), Hippodrome (HIP), Kalabanbougou (KAL), Lafiabougou (LAF), Missabougou (MIS), Niamakoro (NIA), Niaréla (NIAR), Ouolofobougou (OUO), Sabalibougou (SAB), Sotuba (SOT), Sokorodji (SOK), Bacojicoroni (BAC), Médina-Coura (MED), Banconi (BAN). (For interpretation of the references to colour in this figure legend, the reader is referred to the web version of this article.)

The presence of anti-orthohantavirus IgG was detected in 61 of the 413 *R. norvegicus* tested (apparent seroprevalence of 14.8 %). Among immature rats, 3.8 % (*n* = 6/159) were tested seropositive versus 21.7 % (*n* = 55/254) for the mature ones. As SEOV causes persistent infection in rats, the presence of antibodies means that the animal is infected, except in the case of immature individuals, for which the presence of maternal antibodies is possible. Seroprevalence varied between neighborhoods, ranging from 0 % to 29.6 % (Table 1). The distribution of seropositive individuals spanned the entire Bamako district, with the exception of the outlying quarters of Lafiabougou (*n* = 41), Sokorodji (*n* = 12) and Sotuba (SOT) (*n* = 17), where no seropositive brown rats were recorded (Fig. 2).

No *R. rattus* was tested seropositive for anti-*Orthohantavirus* IgG (Table 2).

### 3.4 Orthohantavirus phylogenetic analyses

**Fig. 3:**
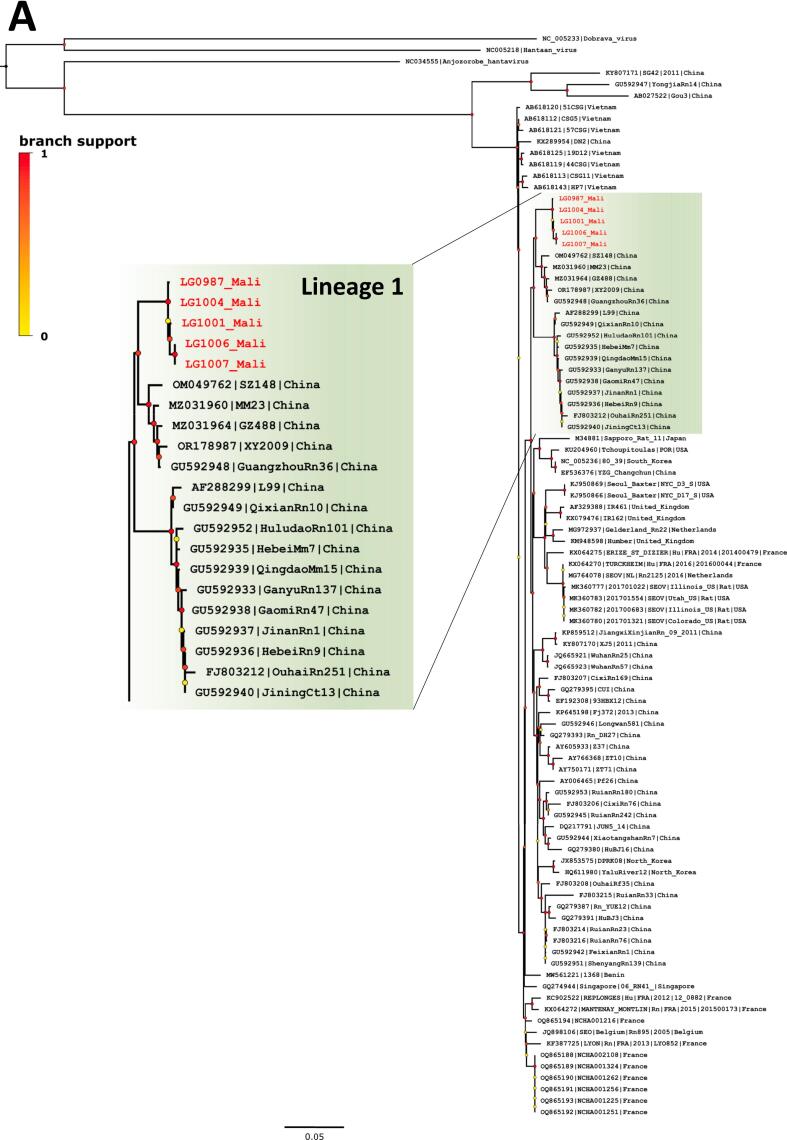

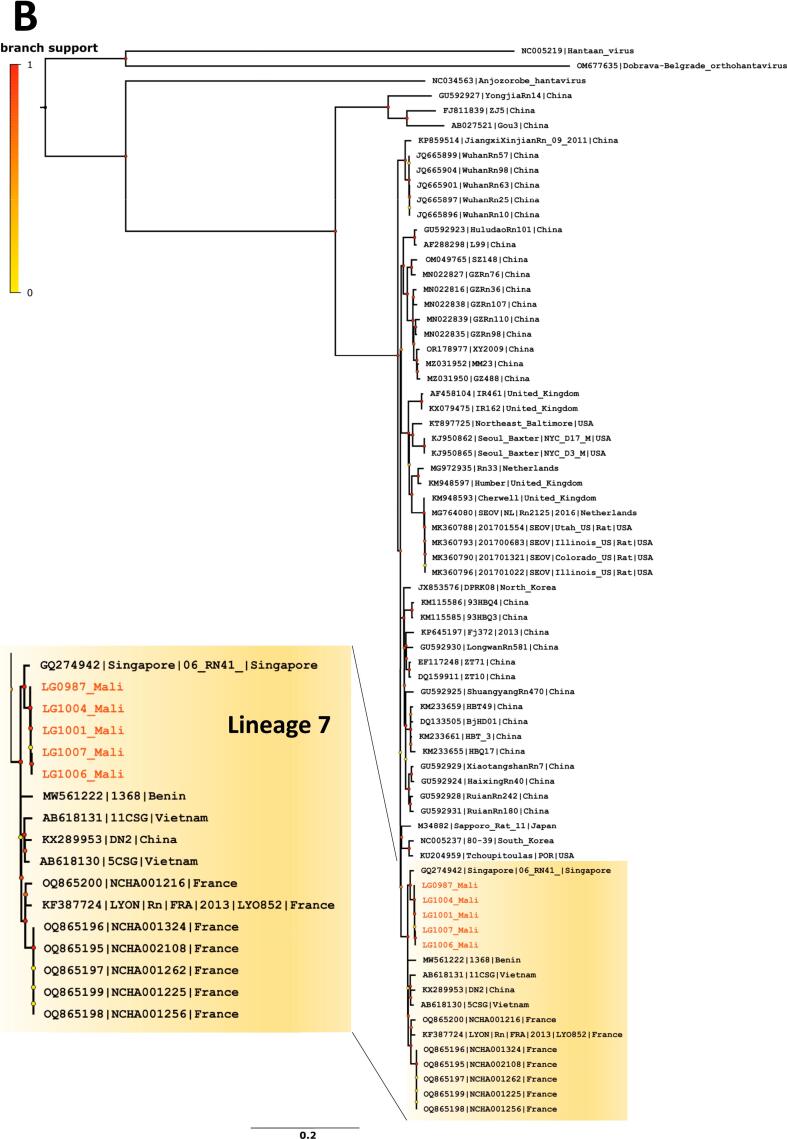

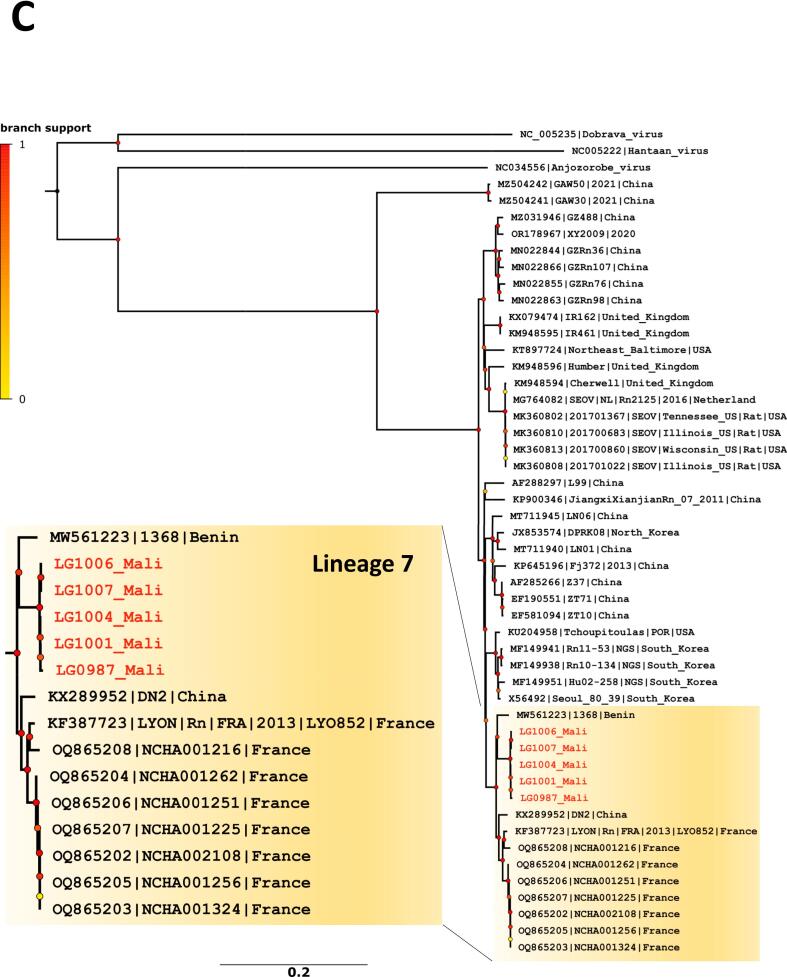
Phylogenetic analyses of Seoul orthohantavirus gene fragments recovered from brown rats trapped in Bamako, Mali (in red) and reference sequences. Phylogenetic trees were generated by the maximum-likelihood method on the complete coding part of the small (A), medium (B) and large (C) segments. Colored point at each node represents branch support as determined by an aLRT test. Scale bars indicate numbers of substitutions per nucleotides. (For interpretation of the references to colour in this figure legend, the reader is referred to the web version of this article.)

Five seropositive *R. norvegicus* with lungs preserved in DNA/RNAShield (four from Fadjiguila and one from Missabougou) were analysed for orthohantavirus characterisation. The specific real-time RT-PCR analysis confirmed that SEOV was the causative agent of these infections (C_T_ values ranged from 22 to 27) and five whole-genome sequences of SEOV were obtained. Phylogenetic analyses produced non concordant phylogenetic topologies for the S, M and L segment datasets. In phylogenetic tree based on S-segment sequences (Fig. 3A), Bamako strains fell with SEOV lineage 1 (as described in [41]) that includes strains from China. It should be noted that a recent study described new strains from the Chinese province of Yunnan, classifying them into a novel subgroup termed ‘NY3’ [42]. The S segment sequences from Mali were found to be most closely related to this subgroup. Besides, phylogenetic trees based on M and L segment sequences (Fig. 3B and C) revealed that Bamako strains fell into the cosmopolitan lineage 7, which includes, among others, strains from France and Benin.

Pairwise comparison of S-, M- and L-segment SEOV sequences of the five strains from Bamako revealed a low level of divergence among them. The maximum nucleotide divergence reached 0.39 % (0 % at the amino-acid level) for S-segment sequences, 0.29 % (0.18 % at the amino-acid level) for M-segment sequences, and 0.44 % (0.23 % at the amino-acid level) for L-segment sequences.

Of the 409 rats tested for both SEOV and leptospires, seven brown rats, trapped in the four neighborhoods with the highest prevalence of leptospires (Table 1), were co-infected.

### 3.5 Analysis of factors influencing pathogen distribution in *R. norvegicus*

Each selected model has been validated through graphical examination of its residuals (Fig. S2 in supplementary material 2). The most parsimonious models included weight as a significant predictor for both SEOV (LRT = 59.41, *p* < 0.001; only mature individuals) and *Leptospira* (LRT = 21.88, p < 0.001) infections, with heavier individuals exhibiting a higher likelihood of infection (Table 3; Figs. S3 & S4 in supplementary material 3 and 4).

**Table 3:** Summary of the most parsimonious Generalized Linear Models (GLMs) finally selected following the model selection procedure. AICc: Akaike's information criterion corrected for finite sample size. Δ: difference between the model selected and the model with the lowest AICc. LRT: Likelihood-ratio test.

Response variable	AICc (Δ)	Predictors selected	LRT	*p*-value
*SEOV*	210.1 (0)	Weight	59.41	1.28e-14
*Leptospira*	167.3 (0)	Weight	21.88	2.896e-06
		Neighborhood	62.52	9.249e-08

For *Leptospira*, the variable “neighborhood” was also kept in the best model (Table 3), and significantly influenced infection probability (LRT = 62.52, p < 0.001).

## 4 Discussion

Pathogenic leptospires were detected in 8 % of brown rats, but only in one black rat, suggesting low prevalence in the latter species in Bamako. This disparity aligns with findings from Benin [25] and may stem from ecological differences. Brown rats, which inhabit ground-level areas near water and sewers where *Leptospira* bacteria can survive during long periods of time [43], are more likely to encounter these bacteria [21]. In contrast, black rats are more regularly found in upper parts of human dwellings, reducing their exposure to water and sewers. It is important to note that other mammal species could host leptospires, potentially serving as additional reservoirs of these pathogens in Bamako [26].

SEOV circulation was detected in brown rats with a citywide seroprevalence reaching approximately 15 %. SEOV was not detected in black rats despite high prevalence in brown rats in the same neighborhoods. Other studies have also found higher seroprevalence of SEOV in brown rats than in black rats when these two species are found in the same environment [[44], [45], [46]]. In Bamako, the abundance of resources in urban environments combined with ecological differences between brown and black rats, might reduce contact between the two species, thereby restricting virus transmission events between them [47]. A lower abundance of *R. rattus* populations, as suggested by the lower trapping success observed compared to *R. norvegicus*, might then not allow the persistence of the virus within this species. Nevertheless, the possibility of SEOV circulation within black rats in Bamako cannot be ruled out. It is possible that the virus circulates at such low levels in this species that the likelihood of capturing infected individuals is minimal, which may explain the absence of SEOV-seropositive individuals in the small sample of black rats trapped in this study.

Only seven co-infections were detected. Coinfected rats were trapped in neighborhoods with the highest prevalence of leptospires, as previously reported [27]. We did not find any evidence of a significant association between the two pathogens; the presence of one did not reliably predict whether the other is present or absent.

Biotic and/or abiotic factors are likely to influence pathogen persistence and transmission. We found that heavier brown rats are more likely to be infected with SEOV and leptospires, which is consistent with other studies [48]. As rodents' weight is an acceptable proxy for age [47], older rats are more likely to have contracted these chronic infections [49]. Pathogenic leptospires exhibited a heterogeneous distribution across Bamako city, with particularly high levels of prevalence (> 20 %) detected in some neighborhoods, notably Bacojicoroni, Médina-Coura, Banconi, and Badalabougou. However, low numbers captured in some neighborhoods might have overlooked leptospires circulation in other areas. Bamako is a heterogeneous urban environment, made up of a mosaic of neighborhoods with different levels of sanitation. Water circulates in many places, such as the Niger river that crosses the city and the numerous wastewater collectors (as in Médina-Coura and Banconi) and river tributaries, which could locally facilitate leptospires' survival and transmission to rodents and humans. Three of the four neighborhoods with highest prevalence (Bacojicoroni, Médina-Coura and Banconi) were sampled during the cool dry season following the rainy season (Table S1). This may have inflated prevalence estimates as rainfall likely facilitates bacterial suspension in water, increasing transmission risks [22]. Badalabougou was sampled during the hot dry season but its immediate proximity to the river (Fig. S1) and its market gardening areas make it a wetter zone, conducive to the circulation of leptospires. A more detailed understanding of how environmental and meteorological factors influence leptospirosis distribution in Bamako is essential to inform prevention and control strategies that will mitigate human exposure risks. Updated maps of surface waters, including the wastewater collector network and Niger River tributaries, are also crucial to better appraise the role of water in leptospires' circulation between the environment, rodent reservoirs, and humans. Lastly, a fine-scale mapping of land use in Bamako is critical to assess the factors that shape human exposure. To date, such data are currently limited to a single study [50] that highlights the dramatic expansion of built-up areas in the city, but is at too coarse a resolution.

In contrast, SEOV was detected in most of the city with, however, inter-neighborhood variations in seroprevalence. The absence of the virus in the city's outskirts (Lafiabougou and Sotuba) may indicate that the virus has not yet spread to these peri-urban areas. In Sokorodji, the small number of brown rats captured (*N* = 12) may also reflect a less abundant population, potentially limiting pathogen establishment. As one of Bamako's most recently urbanized neighborhoods [51], Sokorodji may still be in the early stages of rat colonization or have habitat conditions that are less suitable for *R. norvegicus*. If the limited abundance of brown rats is due to the area's recent establishment rather than habitat characteristics, future rodent dispersal could facilitate the spread of the virus [49].

It is known that wet environment may enhance the survival time of orthohantaviruses, whereas heat and ultraviolet radiation could reduce their infectivity [52]. On the other hand, increased temperatures could also promote aerosol generation leading to higher volumes of small inhalable viral particulates and therefore to virus transmission, between rodents or to humans [53]. Unfortunately, our sampling design did not allow us to test for a season effect here.

Phylogenetic analyses of SEOV strains of Bamako suggested possible inter-lineage reassortment between the cosmopolitan lineage 7, identified in Benin [16] and France [54], and lineage 1, documented in China, three countries with significant trade ties to Mali [55]. However, it remains unclear whether this reassortment could have occurred locally or prior to the strains' introduction to Bamako. Known SEOV variants from Senegal are more phylogenetically distant (lineage 3 or 4) [16] suggesting that Senegal is not the origin of the strains circulating in Bamako. But the limited size of sequences from Senegal complicates phylogenetic reconstructions. In addition, it is also possible that other strains are currently circulating or have circulated in Senegal without being identified.

Prior to this study, SEOV presence was unknown to health services in Bamako, and to our knowledge, the virus was not investigated for patients presenting febrile symptoms. Until now, the only indication of orthohantavirus-induced human illness in Mali came from a retrospective analysis of serum samples collected between 2009 and 2013 in other Malian cities [19], which showed orthohantavirus seroprevalence of 7.2 % (14.4 % for *Leptospira).* More recently, no clear etiology was found in 20.4 % of a cohort of 108 febrile patients from hospitals across Bamako who were tested for various pathogens [32]. In this study (carried out around the same time as ours (2020−2022)), *Leptospira* was not detected and orthohantaviruses were not tested. Given the high prevalence of SEOV in rats in Bamako, it cannot be ruled out that some of these illnesses are due to this virus. Clinican awareness for Orthohantavirus infections and leptospirosis in patients showing non-specific symptoms, including hemorrhagic ones like thrombocytopenia also associated with malaria, is therefore essential [[10], [11], [12],^29^,^56^]. SEOV diagnosis could reduce unnecessary antibiotic use, provide clinicians with better prognostic tools, and inform prevention efforts [10]. The treatment of HFRS being purely symptomatic, prevention of infection by limiting contact with rodents and their excretions, or controlling rodent populations, remains essential. In this last respect, community-led sanitation programs to disrupt transmission cycles should be implemented. This could be part of a more general strategy of ecologically-based rodent management in the city of Bamako, following recent recommendations issued from similar programs conducted in rural contexts [57,58].

## CRediT authorship contribution statement

**J. Garona:** Writing – review & editing, Writing – original draft, Visualization, Investigation, Formal analysis. **A. Berard:** Writing – review & editing, Writing – original draft, Visualization, Investigation, Formal analysis. **C. Tatard:** Writing – review & editing, Investigation. **A. Kwasiborski:** Writing – review & editing, Investigation. **P. Gauthier:** Writing – review & editing, Investigation. **S. Ag Atteynine:** Writing – review & editing, Resources. **V. Hourdel:** Writing – review & editing, Investigation. **A. Eusebe:** Writing – review & editing, Investigation. **C. Diagne:** Writing – review & editing, Validation, Methodology. **V. Caro:** Writing – review & editing, Validation. **C. Brouat:** Writing – review & editing, Validation. **N. Charbonnel:** Writing – review & editing, Supervision. **V. Sauvage:** Writing – review & editing, Validation, Methodology. **L. Granjon:** Writing – review & editing, Supervision, Conceptualization. **G. Castel:** Writing – review & editing, Writing – original draft, Supervision, Conceptualization.

## Declaration of competing interest

The authors declare that they have no known competing financial interests or personal relationships that could have appeared to influence the work reported in this paper.

## Data Availability

All datas are available. Links to the Data are provided in the manuscript. The viral sequences are available in Genbank.

## Appendix A Supplementary data

Supplementary material 1 Map of Bamako showing the different neighborhoods sampled in this study represented by a multi-letter code. Badalabougou (BAD), Bamako Coura (COU), Fadjiguila (FAD), Hippodrome (HIP), Kalabanbougou (KAL), Lafiabougou (LAF), Missabougou (MIS), Niamakoro (NIA), Niaréla (NIAR), Ouolofobougou (OUO), Sabalibougou (SAB), Sotuba (SOT), Sokorodji (SOK), Bacojicoroni (BAC), Médina-Coura (MED), Banconi (BAN). Polygons indicate the associated sampling area. The Niger River is shown in green. GPS coordinate grid is indicated. The map was created using QGis software, and the base map is based on four Sentinel-2 images (01-2024) downloaded from "theia_land.fr.". Supplementary material 1

Supplementary material 2 Residual diagnostics for the most parsimonious Generalized Linear Models (GLMs) explaining the likelihood of infection by SEOV (panel a) and Leptospira (panel b) circulation. This plot displays the diagnostic residuals generalized linear model (GLM) fitted to the dataset. The residuals are simulated from the models using the simulateResiduals function from the ‘DHARMa’ R package, which generates simulated residuals under the null hypothesis of a well-fitting model. Supplementary material 2

Supplementary material 3 Distribution of weight in sexually mature R. norvegicus according to SEOV serological status. Graph generated with R (v4.4.0). Supplementary material 3

Supplementary material 4 Distribution of weight in sexually mature R. norvegicus according to leptospire infection status. Graph generated with R (v4.4.0). Supplementary material 4

Supplementary material 5 . Rattus’ captures in 16 neighborhoods of Bamako city between October 2021 and March 2023. Supplementary material 5

Supplementary material 6 Average climatic values (rainfall, minimal and maximal temperature monthly values in millimeters and degrees Celsius, respectively) of the Rainy (May-October), cool dry (November-February) and hot dry (March - April) seasons in Bamako, between 1991 and 2004. Supplementary material 6
