# Supplementary figures and images for: Detection of two zoonotic pathogens, Seoul orthohantavirus and pathogenic Leptospira, in rats of Bamako, Mali (2021−2023)
Source: One Health. 2025 May 23;20:101085. doi: 10.1016/j.onehlt.2025.101085 (PMC12158529; doi:10.1016/j.onehlt.2025.101085)

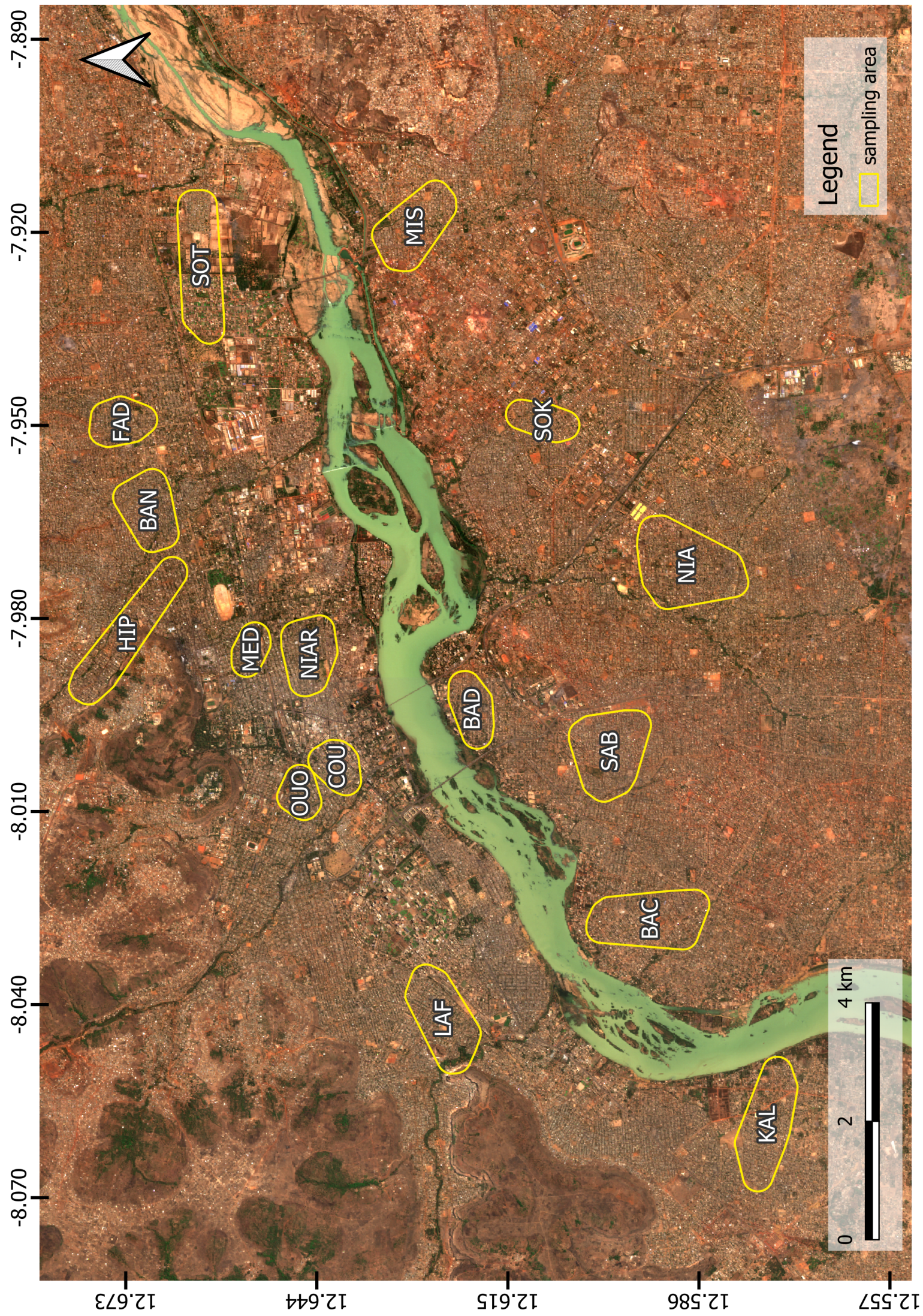

Supplement: Supplementary material 1 — Map of Bamako showing the different neighborhoods sampled in this study represented by a multi-letter code. Badalabougou (BAD), Bamako Coura (COU), Fadjiguila (FAD), Hippodrome (HIP), Kalabanbougou (KAL), Lafiabougou (LAF), Missabougou (MIS), Niamakoro (NIA), Niaréla (NIAR), Ouolofobougou (OUO), Sabalibougou (SAB), Sotuba (SOT), Sokorodji (SOK), Bacojicoroni (BAC), Médina-Coura (MED), Banconi (BAN). Polygons indicate the associated sampling area. The Niger River is shown in green. GPS coordinate grid is indicated. The map was created using QGis software, and the base map is based on four Sentinel-2 images (01-2024) downloaded from "theia_land.fr.". [file mmc1.pdf]

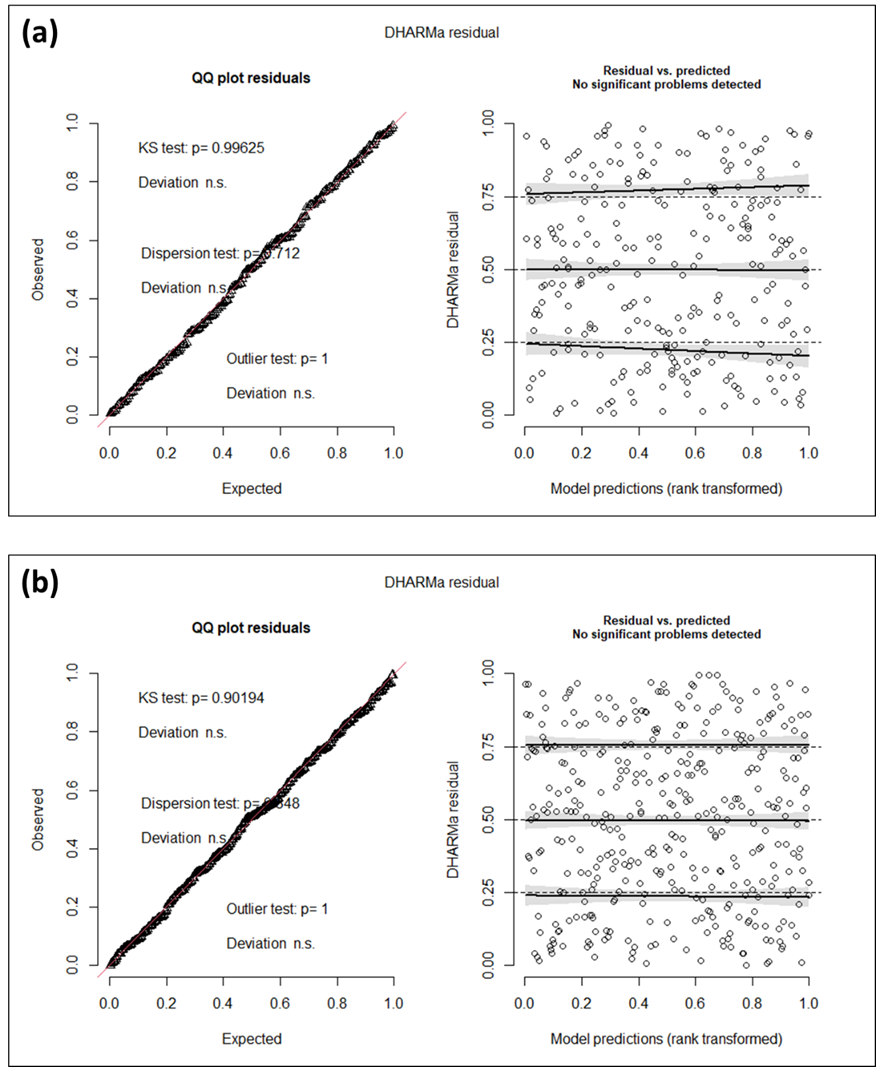

Supplement: Supplementary material 2 — Residual diagnostics for the most parsimonious Generalized Linear Models (GLMs) explaining the likelihood of infection by SEOV (panel a) and Leptospira (panel b) circulation. This plot displays the diagnostic residuals generalized linear model (GLM) fitted to the dataset. The residuals are simulated from the models using the simulateResiduals function from the ‘DHARMa’ R package, which generates simulated residuals under the null hypothesis of a well-fitting model. [file mmc2.docx]

**
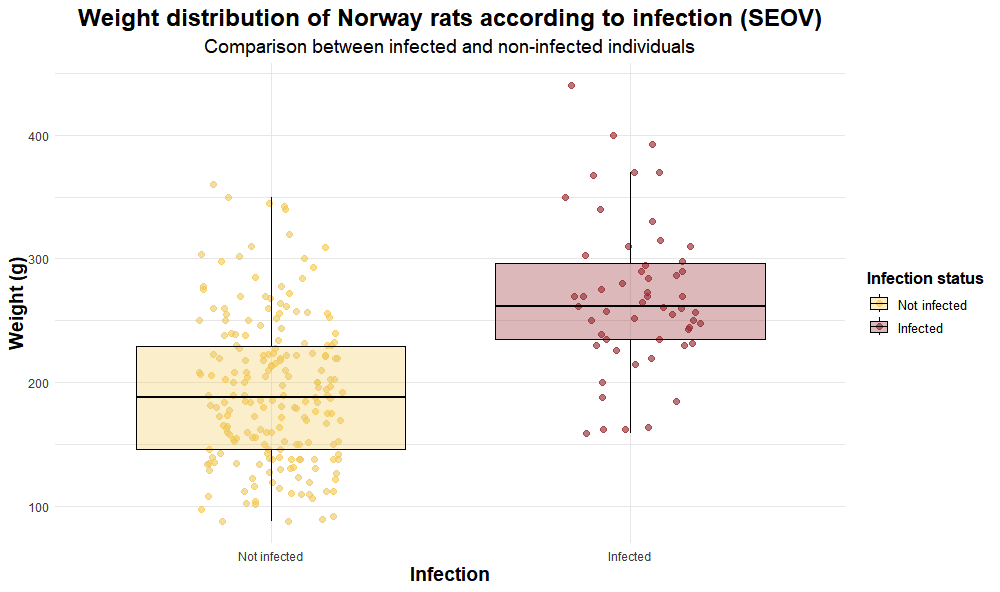
**

Supplement: Supplementary material 3 — Distribution of weight in sexually mature R. norvegicus according to SEOV serological status. Graph generated with R (v4.4.0). [file mmc3.docx]

**
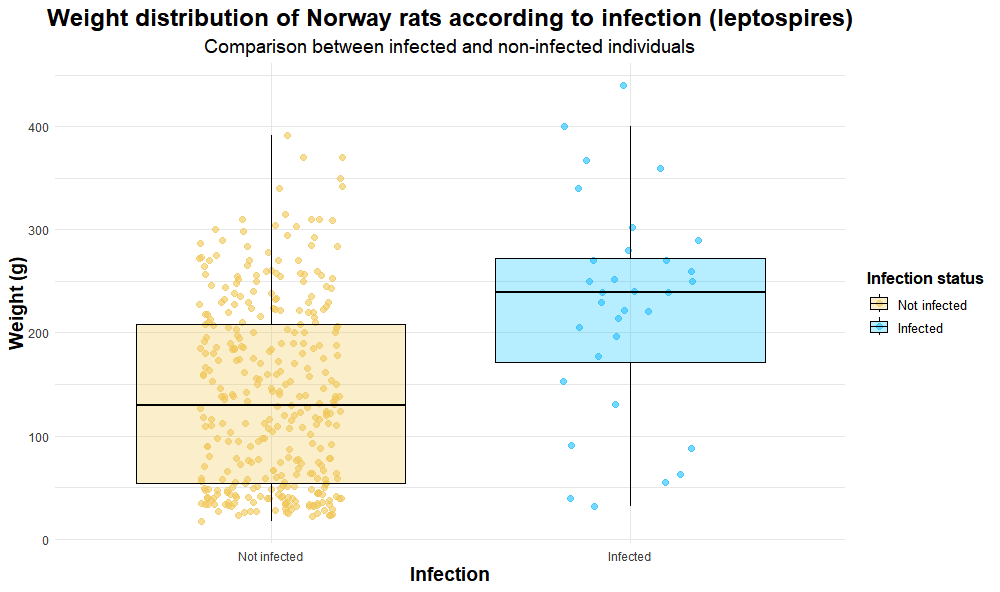
**

Supplement: Supplementary material 4 — Distribution of weight in sexually mature R. norvegicus according to leptospire infection status. Graph generated with R (v4.4.0). [file mmc4.docx]
