# Supplementary material for: Detection of two zoonotic pathogens, Seoul orthohantavirus and pathogenic Leptospira, in rats of Bamako, Mali (2021−2023)
Source: One Health. 2025 May 23;20:101085. doi: 10.1016/j.onehlt.2025.101085 (PMC12158529; doi:10.1016/j.onehlt.2025.101085)
Supplement: Supplementary material 5 — . Rattus’ captures in 16 neighborhoods of Bamako city between October 2021 and March 2023. [file mmc5.docx]

Table S1: Rattus’ captures in 16 neighborhoods of Bamako city between October 2021 and March 2023.

| **Neigborhood (longitude/latitude)** | **Sampling period** | **Season** | **Trap.night number** | ***Rattus norvegicus*** | ***Rattus rattus*** |
| --- | --- | --- | --- | --- | --- |
| Sotuba  (-7.9255/12.6605) | Oct. 2021 | Rainy | 467 | 17 | 3 |
| Fadjiguila  (-7.9495/12.6724) | Mar. 2022 and May 2023 | Hot dry/ Rainy | 563 | 44 | 0 |
| Niaréla  (-7.9855/12.64475) | Nov. 2021 | Cool dry | 480 | 26 | 4 |
| Médina-Coura  (-7.985/12.654) | Jan. 2022 | Cool dry | 142 | 18 | 2 |
| Banconi  (-7.965/12.6695) | Feb. 2022 | Cool dry | 299 | 37 | 0 |
| Hippodrome  (-7.98075/12.6724) | Apr. 2022 | Hot dry | 623 | 23 | 0 |
| Ouolofobougou  (-8.0075/12.647) | Nov. 2021 | Cool dry | 405 | 19 | 10 |
| Bamako Coura  (-8.00178/12.64125) | May 2022 | Rainy | 469 | 27 | 2 |
| Lafiabougou  (-8.0415/12.6245) | Dec. 2021 | Cool dry | 497 | 41 | 0 |
| Kalabanbougou  (-8.0555/12.574) | Oct. 2022 | Rainy | 694 | 13 | 11 |
| Badalabougou  (-7.994/12.62) | Mar. 2022 | Hot dry | 579 | 25 | 0 |
| Bacodjicoroni  (-8.025/12.5905) | Feb. 2023 | Cool dry | 773 | 20 | 8 |
| Sabalibougou  (-8.005/12.6) | Jul. 2022 | Rainy | 621 | 27 | 0 |
| Missabougou  (-7.9185/12.62875) | Mar. 2022 and Apr. 2023 | Hot dry | 607 | 39 | 2 |
| Niamakoro  (-7.972/12.59) | Jun. 2022 | Rainy | 622 | 25 | 0 |
| Sokorodji  (-7.949/12.6115) | Mar. 2023 | Hot dry | 749 | 12 | 0 |
| **Total** |  |  | 8590 | 413 | 42 |
