# Supplementary material for: Detection of two zoonotic pathogens, Seoul orthohantavirus and pathogenic Leptospira, in rats of Bamako, Mali (2021−2023)
Source: One Health. 2025 May 23;20:101085. doi: 10.1016/j.onehlt.2025.101085 (PMC12158529; doi:10.1016/j.onehlt.2025.101085)
Supplement: Supplementary material 6 — Average climatic values (rainfall, minimal and maximal temperature monthly values in millimeters and degrees Celsius, respectively) of the Rainy (May-October), cool dry (November-February) and hot dry (March - April) seasons in Bamako, between 1991 and 2004. [file mmc6.docx]

Table S2 : Average climatic values (rainfall, minimal and maximal temperature monthly values in millimeters and degrees Celsius, respectively) of the Rainy (May-October), cool dry (November-February) and hot dry (March - April) seasons in Bamako, between 1991 and 2004.

|  | **Rainy season** | **Cool dry season** | **Hot dry season** |
| --- | --- | --- | --- |
| **Monthly Rainfall** |  |  |  |
| Mean | 883,2 | 1,0 | 7,0 |
| Min | 561,7 | 0 | 0 |
| Max | 1256,3 | 35 | 53,2 |
| **Monthly Min T°** |  |  |  |
| Mean | 23,1 | 17,5 | 24,2 |
| Min T° | 19 | 12,2 | 20,6 |
| Max | 27,7 | 22,9 | 28,8 |
| **Monthly Max T°** |  |  |  |
| Mean | 33,9 | 34,3 | 39,2 |
| Min T° | 28,6 | 26,8 | 36 |
| Max | 47,6 | 38,8 | 42,2 |

(Data from agroecology unit of the Bamako-Sotuba IER center)
